# Estimating the relationship between fitness and metabolic rate: which rate should we use?

**DOI:** 10.1098/rstb.2022.0491

**Published:** 2024-02-26

**Authors:** Hayley Cameron, Dustin Marshall

**Affiliations:** Centre for Geometric Biology, School of Biological Sciences, Monash University, Clayton, Victoria 3800, Australia

**Keywords:** absolute metabolic rate, evolutionary physiology, performance, mass-independent metabolic rate, metabolic scaling, selection analysis

## Abstract

As physiologists seek to better understand how and why metabolism varies, they have focused on how metabolic rate covaries with fitness—that is, selection. Evolutionary biologists have developed a sophisticated framework for exploring selection, but there are particular challenges associated with estimating selection on metabolic rate owing to its allometric relationship with body mass. Most researchers estimate selection on mass and absolute metabolic rate; or selection on mass and mass-independent metabolic rate (MIMR)—the residuals generated from a nonlinear regression. These approaches are sometimes treated as synonymous: their coefficients are often interpreted in the same way. Here, we show that these approaches are not equivalent because absolute metabolic rate and MIMR are different traits. We also show that it is difficult to make sound biological inferences about selection on absolute metabolic rate because its causal relationship with mass is enigmatic. By contrast, MIMR requires less-desirable statistical practices (i.e. residuals as a predictor), but provides clearer causal pathways. Moreover, we argue that estimates of selection on MIMR have more meaningful interpretations for physiologists interested in the drivers of variation in metabolic allometry.

This article is part of the theme issue ‘The evolutionary significance of variation in metabolic rates’.

## 1. Introduction

Metabolism fascinates biologists for obvious reasons. It is fundamental, because it sets the rate at which organisms can consume resources and do biological work; it is interesting because it varies so much across biological scales (i.e. within-individuals to among species; [1]), and covaries with other components of the life history [2,3]. The august history of ecophysiology has created a wealth of data and theory—it is perhaps one of the best-studied traits in biology, and yet challenges remain.

Physiologists traditionally focused on comparing the metabolic rates of species that varied in size and lifestyle, seeking to understand broad macroevolutionary patterns [4,5]. This approach dominated much of the twentieth century. The focus then shifted to understanding the potential drivers of these patterns, with a strong mechanistic focus. Various attempts were made to reconcile the variation we observe with biophysical principles [6,7]. However, even while there was a strong focus on the use of mechanistic models to explain macroevolutionary patterns, there was a burgeoning discussion of how intraspecific microevolutionary processes might be responsible [8–15]—a viable, if largely overlooked, alternative [16].

**Figure 1.**
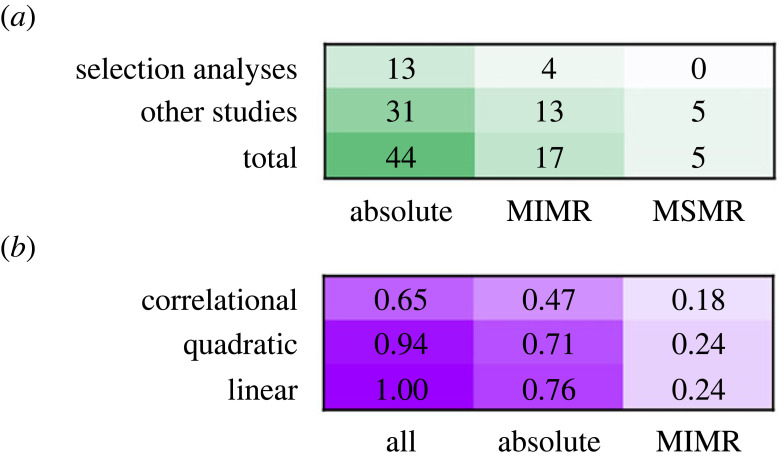
Qualitative literature map of the studies that have estimated the relationship between metabolic rate and fitness (as survival or reproduction; see the electronic supplementary material, methods S1). (*a*) The number of studies that have used different analytic approaches (selection approach or other; as rows) and metrics of metabolic rate (as columns): absolute metabolic rate, mass-independent metabolic rate (MIMR) and mass-specific metabolic rate (MSMR). (*b*) The proportion of studies that have used the Lande & Arnold [17] approach (17 studies total) to quantify selection gradients for the different forms of selection (rows; see box 1 for definitions) using different metabolic traits (columns; first column shows all 17 studies together). In both panels, darker colours indicate that more studies have used a particular approach; lighter colours indicate fewer studies have used that approach.

Meanwhile, the field of evolutionary biology was undergoing a revolution of its own. From the 1980s onwards, formal approaches to estimating selection, a key process in evolution, were developed. The most influential of these approaches was that of Lande & Arnold [17], which provided a way to estimate the strength and form of selection acting on correlated traits as selection gradients (see box 1 for details). In essence this approach offered a way in which biologists could estimate the relationship between traits and fitness in a framework that was directly compatible with evolutionary theory. Since its development, it has become a core approach in evolutionary biology [29,30], and has increasingly been used to understand how metabolism and fitness covary (figure 1 and references therein; electronic supplementary material, methods S1).

Box 1. **A multiple regression approach to estimating selection**

Selection describes the covariance between phenotype and fitness, where the fitness of an individual is determined by its contribution of offspring to the next generation [18]. Given this definition, physiologists seeking to estimate selection on metabolic rate should strive to measure survival and lifetime reproductive output whenever possible [1]. For selection to occur, there must be at least some phenotypic variation in the trait(s) of interest (in our case, metabolism); and variation in fitness among individuals (opportunity for selection). When these traits are heritable, selection will alter the distribution of phenotypes across generations—that is, traits will evolve [18,19].

Selection is most commonly estimated as selection gradients: the coefficients (slopes) obtained from multiple regressions [17]. This approach involves regressing relativized fitness against standardized values of phenotypic trait(s) to obtain standardized estimates of selection that are directly comparable among studies. Phenotypic values are typically standardized to a mean of 0 and variance of 1, and fitness relativized by the mean (but see [20] for discussions about the inferential implications of relativizing within or among environments). Selection can take three general forms: linear, quadratic and correlational, and these may operate in isolation or combination to determine the response to selection. We briefly outline the steps involved in estimating gradients for the different forms of selection below using two traits, but this approach can easily accommodate more traits if necessary.

Directional (or linear) selection occurs when traits (*z_i_*) covary linearly with fitness (*ω*), and is fitted as a linear regression:

1.1 $$\omega =\alpha +\beta_{1}z_{1}+\beta_{2}z_{2}+\varepsilon ,$$

where *α* is the intercept from the regression; *β*_1_ is the linear selection gradient (coefficient) giving the direction and magnitude of selection acting on trait 1; *β*_2_ is the linear selection gradient for trait 2; and *ε* is the error term from the regression. If a trait is sufficiently heritable, and is not constrained by other traits that are also correlated with fitness, persistent positive or negative directional selection (determined by the sign of *β*) should increase or decrease the trait mean of a population, respectively.

By contrast, quadratic selection is a form of nonlinear selection that is defined by a curvilinear relationship between phenotype and fitness. Quadratic selection is fitted by including second-order polynomials in the regression equation:

1.2 $$\omega =\alpha +\beta_{1}z_{1}+\beta_{2}z_{2}+\gamma_{1}z_{1}^{2}+\gamma_{2}z_{2}2+\varepsilon ,$$

where *γ*_1_ is the quadratic selection gradient (coefficient) giving the direction and magnitude of quadratic selection acting on trait 1; and *γ*_2_ is the quadratic selection gradient for trait 2 (other terms are defined above). Note that quadratic selection gradients (and their errors) must be doubled [21]. Under the special case that quadratic selection occurs without any directional selection (i.e. *β* = 0), quadratic selection is said to be stabilizing (when *γ* is negative) or disruptive (when *γ* is positive); otherwise these are referred to as concave or convex selection, respectively. Stabilizing (or concave) selection occurs when fitness is maximized at a single trait value and extreme phenotypes are disfavoured: thus, evolution should decrease phenotypic variance in a population. By contrast, disruptive (convex) selection favours extreme phenotypes, such that phenotypic variance should increase.

Another form of nonlinear selection is correlational selection, which occurs when combinations of two traits interact to affect fitness, and is estimated by the cross-product coefficient (*δ*), or interaction term, between two traits in the multiple regression:

1.3 $$\omega =\alpha +\beta_{1}z_{1}+\beta_{2}z_{2}+\gamma_{1}z_{1}^{2}+\gamma_{2}z_{2}2+\delta z_{1},z_{2}+\varepsilon .$$

Correlational selection alters trait correlations across generations, and again its coefficient (*δ*) can be positive or negative. Positive correlational selection occurs when higher values of both traits (and lower values of both traits) yield higher fitness, such that positive covariances between these traits should evolve. By contrast, negative correlational selection occurs when fitness is greater for combinations of high and low trait values, such that selection should generate negative covariances between traits. It is worth noting that relatively fewer studies estimate correlational selection (cf. linear and quadratic selection: see [22] and figure 1*b*). Correlational selection between mass and metabolism can yield intriguing insights about the evolutionary drivers of metabolic allometry [1], however, and we join others in encouraging that experimenters explore this form of selection more often in future studies [23].

Lande & Arnold [17] showed that ordinary least-squared (OLS) regression reliably estimates selection gradients irrespective of the underlying distribution of fitness or phenotypic traits—but testing the statistical significance of these gradients with an OLS approach is sometimes inappropriate [24]. As such, selection gradients are typically estimated from OLS regressions, while statistical tests are performed using generalized linear models that most appropriately describe the distribution of the underlying data [25,26].

Both parameter estimation of selection gradients, and their statistical tests, are performed via sequential model building (see [27] for a detailed guide). To begin, one estimates baseline performance in an intercept only model, then sequentially adds additional terms that describe the different forms of selection: starting with linear, then quadratic and finally correlational selection (as demonstrated in equations (1.1)–(1.3)). At each step, the significance of selection is determined by comparing the fit of the subsequent model to its predecessor using likelihood ratio tests. We note that this approach can also be expanded to test for differences in selection among environments, and we refer readers to [27,28] for details.

We are excited about the growth of studies that seek to formally estimate selection on metabolism, but there are some issues to be considered when applying this framework to metabolic rate. We recommend Pettersen *et al*. [23] as a starting point for those interested in why we might wish to integrate microevolutionary theory with physiology more generally. Below we explore the challenges associated with estimating selection as it pertains to metabolic rate and mass; and provide some guidance on the pros and cons of different approaches.

## 2. The problem: describing selection on body mass and metabolic rate

**Figure 2.**
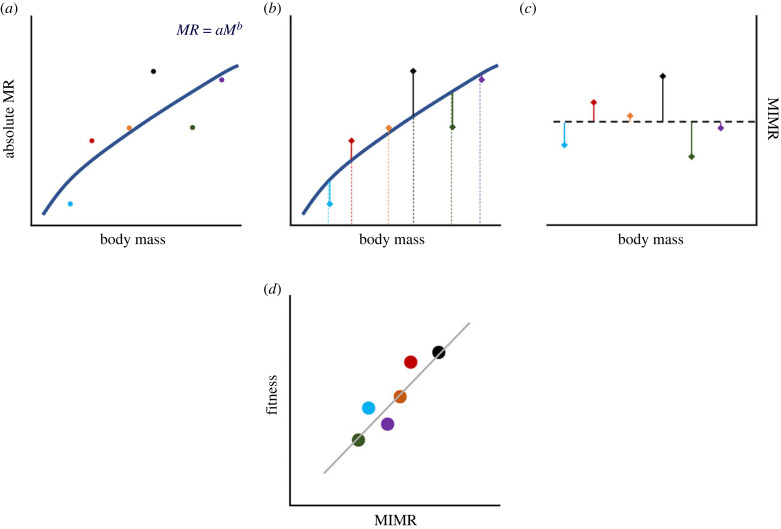
A schematic of the approach for extracting values of MIMR to use as trait values in a selection analysis. (*a*) shows a hypothetical allometric relationship (solid navy line) between absolute metabolic rate (MR) and body mass (M), where each colour represents data from a different individual. The exponent (*b*) that describes the slope of this relationship represents metabolic scaling; while the intercept (*a*) represents metabolic level—the descriptor of the average MIMR of the population. (*b*) The residual deviation (thin solid lines) in the absolute MR of each individual (the coloured points) from the overall allometric relationship (solid navy line). The schematic shows how the absolute MR of individuals (coloured dots) can be decomposed into mass-independent (i.e. the residuals; thin solid lines) and mass-dependent (coloured dotted lines) components. (*c*) The residuals extracted from the allometric relationship shown in the previous panels—these residuals provide estimates of MIMR. Note that these are plotted relative to the average MR for an individual of a given body mass (i.e. MIMR values of 0; black dotted line). (*d*) shows how those estimates of MIMR obtained from the preceding panels can then be plotted against fitness to estimate selection on MIMR (here presented as a hypothetical linear relationship that describes positive directional selection on MR: see box 1 for definition).

Metabolism is the cumulative product of various biochemical processes that ultimately combine to determine the rate at which organisms power their biological work. Metabolism therefore depends strongly on body mass—the greater the mass of the organism, the greater the sum total of metabolic processes required to sustain it [1,3]. Considerations of metabolic rate usually include implicit or explicit considerations about body mass. The traditional framework for describing the relationship between mass and metabolism is metabolic scaling [31]: which estimates the exponent of the allometric (nonlinear) relationship between mass and metabolic rate; and ‘metabolic level', the coefficient of the power function that describes the mass-independent component of metabolic rate (figure 2*a* in box 2). Together, metabolic level, metabolic scaling and body mass describe most of the variation in absolute metabolic rate.

Box 2. **Calculating mass-independent metabolic rate: nonlinear versus log–log linear regressions**

**Figure 3.**
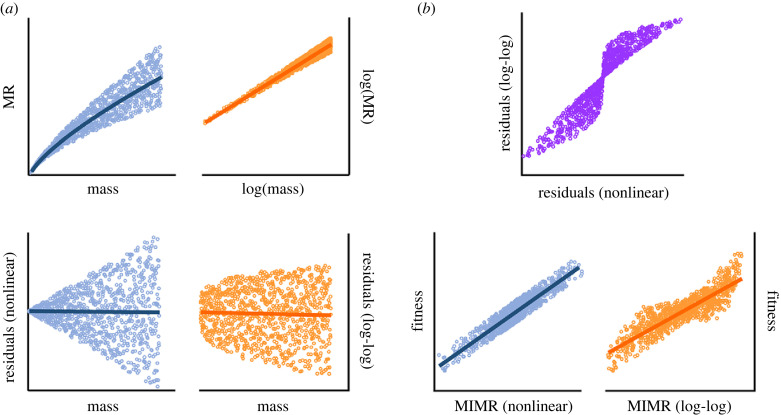
(*a*) Schematic showing how the residuals obtained from the same simulated relationship between mass and MR differ when calculated on a natural scale using nonlinear regressions (blue), or from analyses performed on a log–log scale (orange). (*b*) Residuals calculated from log–log analyses systematically mis-estimate those obtained on a natural scale (in purple). Hence the relationship between values of MIMR and fitness is artificially noisier when residuals are calculated from log–log (orange) relative to nonlinear (blue) analyses.

We advocate for those interested in studying selection on metabolic rate to first calculate mass-independent metabolic rate (MIMR). Here, we briefly walk through the procedure for calculating MIMR (figure 2), and illustrate why a nonlinear regression approach is best (figure 3; see also the electronic supplementary material, methods S1). Consider the realistic, but hypothetical, allometric relationship between body mass and metabolic rate shown in figure 2*a*. Each coloured point represents a different individual. While the general relationship between mass and metabolism is well represented by the blue line where metabolic rate scales with body mass at *b* = 0.75, individuals deviate from that overall relationship (figure 2*b*). For example, the red dot indicates an individual that has a higher metabolic rate than would be expected based on its mass (i.e. a positive value of MIMR), whereas the green individual has a lower metabolic rate than would be expected (i.e. a negative value of MIMR). After fitting a nonlinear regression, we can easily extract the residuals for each individual (figure 2*b*). Those residuals represent MIMR and importantly, those values show no covariance with body mass (figure 2*c*). We can then take those MIMR values and relate them to the fitness of those individuals, shown in this case as a simple positive linear relationship between MIMR and fitness (figure 2*d*).

When using this residual approach to estimate MIMR, it might be tempting to fit a log–log analysis with a linear regression rather than a nonlinear regression in natural space (figure 3). Log–log analyses have the virtue of allowing linear models and are useful for counteracting heteroscedacity (figure 3*a*). Using a log–log analysis to generate residuals is inappropriate, however, because residual deviance in log–log space will depend on *x* (in this case body mass), such that two individuals with the same residual value in log–log space but very different body masses, will have different residual values when MIMR is calculated in natural (nonlinear) space (though these will be in same direction). The top panel in figure 3*b* shows the relationship between MIMR values estimated using residuals from a nonlinear (shown on the *x*-axis) and log–log linear regression (shown on the *y*-axis). As shown, many of these residual values show no relationship. In particular, small deviations from residuals of zero are exaggerated, and extreme values of residuals are diminished, when extracted from a log–log (relative to nonlinear) analysis. Consequently, if we consider a scenario where there is a simple, positive linear relationship between MIMR and fitness, using residuals from a log–log linear regression results in a much poorer fit than those obtained from a nonlinear regression (figure 3*b*). Overall then, we recommend extracting MIMR values by calculating the residuals from nonlinear regressions.

It is difficult to think about how selection acts on metabolism without also considering mass. For example, has metabolism actually evolved if a population responds to selection for increased body mass alone? On the one hand, the mean metabolic rate of the population is likely to increase through the indirect effects of body mass on metabolism. On the other hand, neither scaling nor level have changed: thus, our standard descriptors of metabolism also remain unchanged. From this perspective, most physiologists would probably regard the metabolic phenotype to be the same after the evolutionary change in mass. However, metabolic level and scaling are population-level parameters, not individual traits; whereas selection analyses seek to link the traits of individuals to their performance (see box 1 for details). When seeking to measure the phenotypic covariance between fitness and individual traits, the question then becomes, which traits should one use to test hypotheses about how selection shapes metabolic scaling and level?

Were mass not to vary at all, the identification of the appropriate metabolic trait would be simple: we could just measure selection on absolute metabolic rate. But mass always varies within species, at least a little, and is also a strong determinant of fitness [30]. As such, describing the fitness effects of metabolic rate and body mass becomes more complex. Absolute metabolic rate actually contains at least two components: the metabolic rate that is entirely attributable (and is thus synonymous with) body mass; *and* an extra component that occurs independently of the relationship with mass (figure 2*b* in box 2). Both of these traits are interesting, but we would argue that distinguishing between them is essential in order to make reasonable inferences about how metabolism will evolve.

To illustrate, evolutionary physiologists typically want to understand why metabolism varies among individuals, populations or species, after accounting for differences in body mass. That is, physiologists are specifically interested in the drivers of variation in metabolic level, and hence selection on mass-independent metabolic rate. In other words, we are not usually interested in why a whale has an *absolutely* higher metabolic rate than a mouse—greater masses mean greater total metabolic work [1]. However, physiologists are interested in the evolutionary processes that led to whales having *relatively* lower metabolic rates than mice. That is, they are also interested in the evolutionary drivers of metabolic scaling: how mass, mass-independent metabolism and their covariance affect fitness [32]. On balance therefore, most physiologists are specifically interested in understanding the drivers of evolution in metabolic rate while also considering mass. At least three different approaches have been used to achieve this goal (figure 1).

One approach to account for the effects of mass on metabolism is to simply divide metabolic rate by mass—a metric called mass-specific metabolic rate (MSMR). MSMR has been widely criticized as it does not estimate metabolic rate relative to mass well when the relationship between mass and metabolism is not isometric [33–35], which is a rare case. Fortunately, few studies have used MSMR to investigate the relationship between metabolic rate and fitness (figure 1*a*), and we recommend that this approach be abandoned in future studies.

Alternatively, values of mass-independent metabolic rate (MIMR) can be obtained by extracting the residuals from a nonlinear regression of metabolic rate and body mass ([36]; see box 2 for details). This approach yields values of MIMR that span from positive to negative: positive values indicate an individual has a higher than average metabolic rate for its mass; negative values indicate the converse. This method is intuitively attractive, since it provides an indication of whether metabolic rate is higher or lower than expected based on allometry alone. As we will argue, we believe MIMR provides the greatest congruence between how physiologists think about mass-independent metabolic rate and how the trait is derived.

Most studies attempting to disentangle the fitness effects of body mass from metabolism have estimated selection either on absolute metabolic rate or MIMR (figure 1): and of both, absolute metabolic rate is used most frequently. Researchers tend to interpret findings from these two approaches in the same way: for example, positive linear selection on absolute metabolic rate is interpreted as equivalent to positive linear selection on MIMR (see the electronic supplementary material, references in table S2 and methods). Given the dominance of these two approaches in the literature, and the tendency for researchers to think of them as providing equivalent information, it is important to verify whether they should actually be interpreted in the same way.

**Figure 4.**
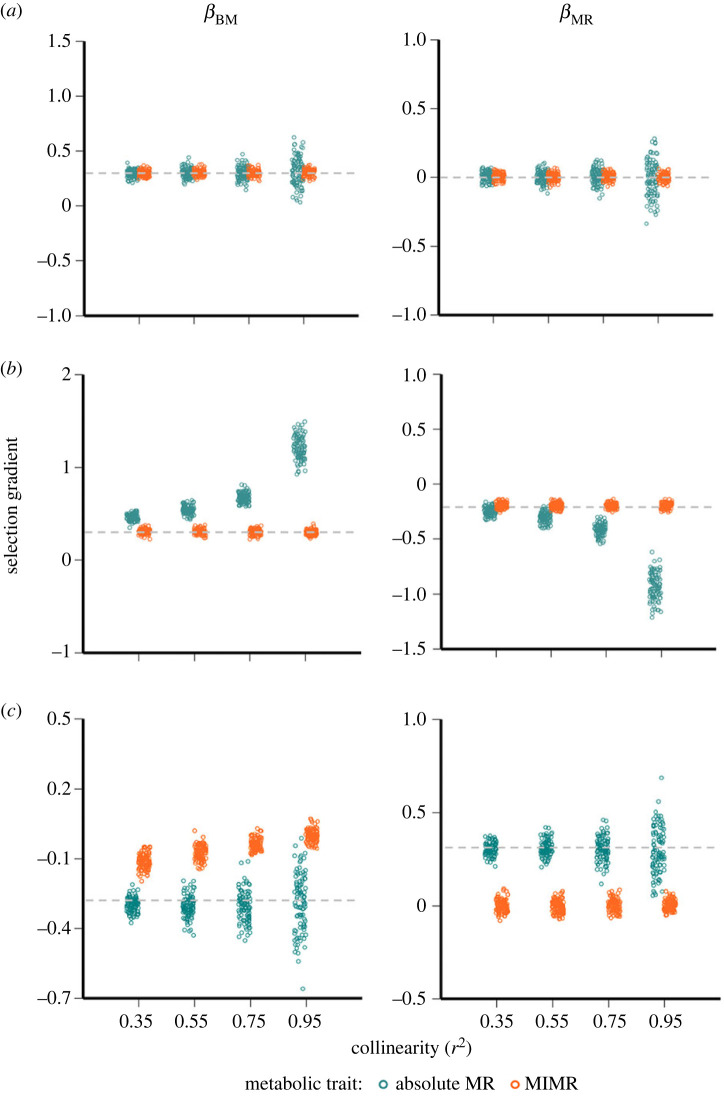
Distribution of the estimates of linear selection gradients for body mass (*β*_BM_) and metabolic rate (*β*_MR_) obtained from multiple linear regressions that used different metrics for metabolic rate: MIMR (orange) or absolute metabolic rate (teal). Plotted estimates are from 100 simulations across the four levels of collinearity (*r*^2^) in the allometric relationship between mass and absolute metabolic rate under three different scenarios of selection: (*a*) positive linear selection on body mass only; (*b*) positive linear selection on body mass and negative linear selection on MIMR, or (*c*) negative linear selection on body mass and positive linear selection on absolute metabolic rate. Grey dotted line shows the simulated value of the selection gradients on the traits of interest in each scenario (see the electronic supplementary material, methods S1 for details and data in S2).

Figure 4 shows that for identical underlying fitness relationships, the estimates of selection on body mass and absolute metabolic rate differ from those on MIMR and mass, particularly when body mass and absolute metabolic rate are highly collinear (see the electronic supplementary material, methods, for details of our simulation approach). The estimates of selection can even differ in sign between the two approaches (figure 4*c,d*). Importantly then, these two approaches are not synonymous, and their results should therefore not be interpreted as such. Similar conclusions have been drawn from analogous studies of relative versus absolute brain size [37].

Note that this difference in coefficients between the approaches (despite the same fitness relationships) does not mean that either approach is inherently wrong or biased: rather, these differences arise because they estimate selection on two different traits [38]. Thus counterintuitively, a study that finds strong *positive* linear selection on both body mass and MIMR actually finds the same result as a study that reports strong *negative* linear selection on body mass and positive linear selection on absolute metabolic rate (figure 4). It is therefore inappropriate to regard these two approaches as interchangeable—two studies could find similar coefficient estimates for each metabolic trait, but the relationships between fitness, mass and metabolism could be different between each study. Thus, the field should at least recognize that the different approaches produce different estimates, and perhaps ideally settle on one particular approach. We will deal with the pros and cons of each approach below so that researchers can make informed choices about which traits to analyse in the future.

## 3. Mass-independent metabolic rate versus absolute metabolic rate: statistical rigour versus biological utility

Using MIMR as a phenotypic trait within a selection framework means we are using a statistical output (residuals) from one analysis as an input for another. Within the literature, there is a view that using residuals is suboptimal and should not be favoured [39,40]. Much of this debate pertains to the use of residuals as dependent variables in downstream analyses, and are irrelevant to the specific use we advocate for here. Nevertheless, there are still statistical and practical downsides to this approach that are worth highlighting.

First, we acknowledge that using residuals as trait values of MIMR essentially gives primacy to the effect of mass (or entirely mass-dependent metabolism) on fitness [41]. We think accounting for mass in this way is appropriate given there are well-established relationships between size and mortality rate, and size and fecundity [42,43]—larger individuals often have higher fitness than smaller conspecifics. Physiologists tend to be interested in whether metabolism affects fitness over and above the effects of body mass. Nevertheless, this primacy should be acknowledged.

Second, calculating MIMR as the residuals from the relationship between body mass and absolute metabolic rate means that MIMR contains multiple components. It contains the true variation in metabolism independent of body mass (the trait we are interested in); as well as measurement error [44,45]; and error generated from the statistical estimation procedure used to obtain these residuals. Yet using MIMR in a selection analysis assumes that all of the variation is biologically ‘real'. Importantly, this error is not propagated through the selection analysis, and poses a non-trivial issue: all else being equal, this additional, unquantified error will result in the systematic under-estimation of selection coefficients [22], and increases the risk of Type II errors in their statistical tests.

Minimizing measurement error therefore takes on new importance when residuals (i.e. MIMR) are included as a predictor in selection analyses. One way to minimize measurement error is by taking repeated measures of metabolic rate for each individual (technical replicates). While technical replication may seem onerous, advances in high-throughput metabolic phenotyping have made this more accessible: thousands of independent metabolic rates can be measured within just a few days [46–48]. These technical replicates can then be incorporated into selection analyses to better decompose MIMR into error and ‘true' MIMR. While a common approach is to average technical replicates and use means as trait values [49], others have shown this approach is insufficient for correcting error-generated biases in selection gradients [22,50]. Rather, we advocate that technical replicates of metabolic rate first be converted into multiple estimates of MIMR for each individual in the dataset; and these then be incorporated into selection analyses using more advanced techniques such as multivariate mixed-effects models [51], or error-in-variables approaches [22,50]. While error-in-variables approaches provide more robust estimates of selection, they are also computationally challenging; thus, mixed-effects models may be a more pragmatic solution [22]. Importantly, both approaches are compatible with a Bayesian framework, and so provide solutions for propagating uncertainty in the estimated parameters [52,53].

Using absolute metabolic rate avoids some of the problems described above, and for these reasons alone, one might favour it. However, absolute metabolic rate also has its issues. First, when body mass and absolute metabolic rate are highly collinear, coefficient estimates tend to be less precise (figure 4). As Morrissey & Ruxton [38] have pointed out, these estimates are still unbiased, and on this basis, argued that collinear predictors are a non-problem. Nevertheless, analysing collinear predictors does result in less precise estimates, such that Type II errors are more likely when interpreting their statistical tests [24,41]. While that imprecision does not generate models that are wrong on average [38], pragmatically it does mean that any one estimate of selection is less likely to represent the underlying biology, so researchers may infer that selection is weaker than it actually is. Morrissey & Ruxton [38] argue that imprecise coefficient estimates are only a problem if one bases model selection procedures on these estimates—pragmatically, researchers tend to make conclusions based on the significance of coefficient estimates so, to us at least, imprecise estimates remain a non-trivial problem.

Trait collinearity may also be problematic given that one is often interested in using selection coefficients in a predictive framework—the goal is to predict how traits will change owing to selection [18,19]. The predictive capacity of models that contain highly collinear traits is contingent upon this collinearity remaining unchanged from one generation to the next [24]. There are clear paths by which selection is expected to alter covariances (collinearity) between traits ([17–19]; see also box 1). Hence predictions based on collinearities that change are less reliable. Whether these downsides are sufficient to avoid the use of absolute metabolic rate is debatable; but they should at least be considered. For further discussion of collinear traits in the context of selection, Mitchell-Old & Shaw [24] and Morrissey & Ruxton [38] are, in our view, a useful place to start.

Ultimately, we favour the use of MIMR over absolute metabolic rate not because of the statistical issues associated with either approach—in fact, on balance, MIMR may be less desirable from a statistical perspective. Rather, we favour MIMR because we believe it is ultimately the biological trait of interest that physiologists are seeking to understand. Selection on MIMR has an intuitive and direct interpretation. For example, assuming there is sufficient genetic (co)variation [51], negative directional selection on MIMR will decrease metabolic level and increase metabolic scaling slightly. This change in both level and scaling is counterintuitive but occurs because shifts in the mean value of *y* (in this case, metabolic rate) changes both the coefficient and exponent of any power function. Likewise, stabilizing selection on MIMR implies that there are strong fitness penalties for having a substantial MIMR—individuals with metabolic rates that are strictly mass dependent will have higher fitness, which may imply variation in metabolic rate is constrained to show strict allometric relationships. These are intuitive interpretations.

**Figure 5.**
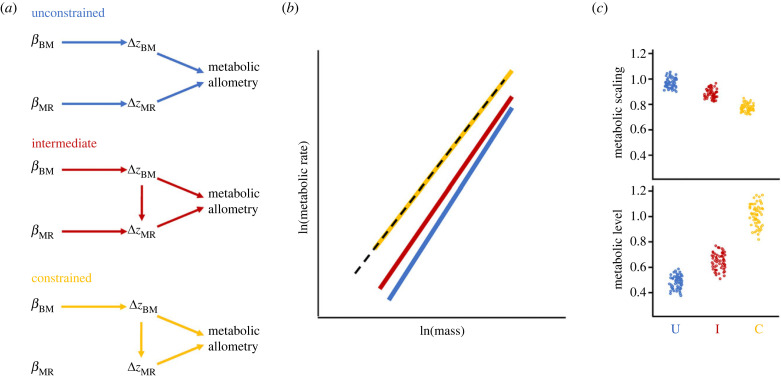
Predicting evolutionary responses to selection on absolute metabolic rate (MR) and body mass (BM). (*a*) Directed acyclic graphs of potential causal relationships between linear selection on the traits of interest (indicated by *β*), changes in the mean trait values (indicated by *ΔZ*) and their ultimate influence on metabolic allometry. The ‘unconstrained' scenario represents the causal pathways by which each trait changes in the absence of any physiological constraint (blue). The ‘constrained’ scenario represents the causal pathways by which metabolism can only change as a function of mass (yellow). The ‘intermediate' scenario represents when both pathways operate (red). (*b*) shows an illustrative example of how different causal pathways can yield different evolutionary outcomes on allometry, given the same magnitude of selection (plotted changes in allometry are from a single simulation run; see the electronic supplementary material, methods S1 and data in S3). The dotted black line represents the allometric relationship between mass and metabolism for generation 1, the different coloured lines represent the allometric relationship after selection in generation 2 according to three causal pathways shown in panel (*a*). (*c*) The outcomes of 100 simulations, showing the impacts of different causal pathways on the predicted responses to selection for metabolic scaling (top right) and level (bottom right). The constrained pathway (C; yellow) retains the original metabolic scaling and level from generation 1. The unconstrained pathway (U: blue) predicts a higher metabolic scaling and lower metabolic level. The intermediate pathway (I: red) sits between these two extremes. Overall, the figure shows that when absolute MR and BM are considered in selection analyses, the underlying causal pathway is unknowable, so it is difficult to make reasonable inferences about how metabolic allometry will evolve.

By contrast, were one to only estimate selection on absolute metabolic rate and mass, one's capacity to make reasonable biological inferences is more limited. Absolute metabolic rate provides no information about the relative proportion of metabolism that is mass-dependent or mass-independent. This limits our ability to make inferences about the causal pathways through which absolute metabolic rate affects fitness, as well as how selection on absolute metabolic rate will alter metabolic allometry ([54]; see also figure 5). To illustrate, consider three scenarios through which selection on absolute metabolic rate can causally alter metabolic allometry: we have deliberately made them coarse for heuristic purposes. In the first scenario, metabolic rate and mass are two distinct traits just like any others—they each undergo evolutionary change according to selection and their genetic (co)variance (figure 5*a*). Under this scenario, there is no causal relationship between mass and metabolic rate—both are free to vary completely independently. Alternatively, metabolic rate could be entirely a function of mass—variation in metabolic rate is completely driven by the allometric relationship, thus even if metabolic rate has some relationship with fitness, it cannot evolve independently of mass. The first scenario views drivers of metabolic rate entirely through a quantitative genetics lens, while the second treats metabolic rate as a physiologically constrained trait: neither is likely, but both are possible. A third, more realistic scenario is intermediate to these extremes—mass has a strong effect on metabolism but selection can alter metabolism independently, at least to some extent. Assuming there is sufficient genetic variation in both mass and metabolism in all three scenarios, we can explore simple predictions of how mass and metabolism might change across generations (see the electronic supplementary material, methods S1 for details of our approach).

We considered an extremely simplified case of linear selection for increased mass and decreased absolute metabolic rate with abundant genetic variation in each trait (and assuming no genetic correlation between them). We find that for the ‘no constraint' scenario, metabolic level is predicted to decrease, and metabolic scaling is predicted to increase (figure 5*b,c*). While for the ‘constrained' scenario, we predict no change in scaling nor level. Evolutionary responses in the intermediate scenario sit between these two extremes. The degree to which metabolic rate is actually constrained to vary with mass thus strongly determines the evolutionary dynamics of metabolic allometry: the same selection will yield very different predictions of how these traits will evolve depending on our assumptions regarding causal pathways.

Physiologists seek to understand the drivers of metabolic allometry, but unfortunately, the extent to which metabolism is (un)constrained by mass is unknowable using an absolute metabolic rate approach. We need to estimate MIMR to access this information. Inferences based on selection analyses of mass and absolute metabolism are therefore less accessible, in our view, because of the ‘black box’ that is the causal relationship between mass and absolute metabolism (figure 5*a*). More prosaically, it is harder to think about absolute metabolism and mass simultaneously and make reasonable inferences about their dynamics when their relative mass-(in)independent components remain enigmatic. These difficulties are only compounded were one to think about more complex scenarios involving nonlinear selection (see box 1 for definitions). By contrast, we can make more straightforward inferences from estimates of selection on MIMR: this approach explicitly acknowledges the synonymous nature of mass and mass-dependent metabolism, and delineates these from MIMR, with an unambiguous causal pathway. Selection on these components together permits clear and simple predictions about the metabolic allometries we seek to understand.

## 4. Conclusion

Overall, we believe that MIMR represents a valid and pragmatic solution to estimating selection on the various components of metabolic rate while accounting for mass. We believe that this metric reveals the otherwise-hidden physiological information of interest [55,56]. Others have also highlighted the evolutionary significance of residual phenotypic vicariance, particularly as it relates to plasticity in behaviour, personality and physiology [56,57]. We agree that such residual within-individual variation is interesting, and is particularly relevant to understanding the evolution of plasticity in metabolic rate [58]. Here, we argue that there is also biologically meaningful information in the residual variance of metabolic allometry (i.e. MIMR)—and that this too, represents a component of phenotypic variance that is heritable and may itself evolve [59,60]. In other fields, researchers have similarly advocated for using residuals as predictors under specific scenarios when they provide better access to the traits of interest, despite the statistical downsides [37,41,61]. However, we recognize that others may take a different view. Regardless, we would suggest that researchers keep clear in their minds that estimates of selection based on different metabolic traits are not synonymous, and require different biological interpretations.

We hope to have provided an accessible guide for robust estimates of selection on metabolic rate that better integrates the traditions of metabolic physiology with formal quantitative genetics. It seems to us that we are entering an exciting time for evolutionary physiology, where technological innovations allow estimates of metabolic rates at unprecedented scales (e.g. [46–48]). The convergence of this technology with sophisticated analytical tools from evolutionary biology [22,50,51] will allow for new and important insights into the evolutionary causes and consequences of variation in metabolic rate.

## Data Availability

The data are provided in the electronic supplementary material [62].
